# Annotations

**Published:** 1887-11-26

**Authors:** 


### Annotations.

**The Science of Superstition.**

Extremes meet. From one idea to its apparent opposite is often but a step, and unimaginative materialism joins hands with superstition and credulity. Such, at least, is the impression one receives after perusing the manual of animal magnetism lately written by MM. Alfred Binet and Charles Féré. This deals in a scientific spirit with those phenomena which people who witness them call miraculous, and those who don't, pronounce incredible. When anyone, even so able a man and acute an observer as Laurence Oliphant, tells us that he saw a man swallow live coals or pass a skewer through his cheek without sign of pain, and even with the appearance of enjoyment, we are inclined to say at once that we do not believe what he says. He goes on his way, we on ours; each despising the other's intellect. But the new explanation offered by the students of animal magnetism is satisfying, at least to those of us who, while we think that any supernatural suspension of natural law is in the highest degree improbable, are willing to admit that apparent interruptions of the action of known laws may result from the presence of some natural law as yet not fully investigated. According to MM. Binet and Féré, the seeming miracle lies, not in the performer, but, like beauty, in the eye of the beholder, who is hypnotised; that is, thrown into a trance, in which he will see things, not as they are, but as the hypnotiser declares them to be. If the subject is told that on awakening from the trance he or she—it is usually she—will not see a certain member of the company present, the thing happens as predicted. She does not see the individual in question. She talks as if he were absent, and it may be in a fashion she would not use if he were near. If she comes in contact with him, she will cry out with the horror we all feel at striking an invisible obstacle. Or if she is told that someone else is a cat or a dog, she will treat him as such, showing her love or dislike for the animal in question. Moreover, for some time, even several days, after awaking from the trance, any suggestion of them will bring back to her the hypnotic delusions. This, doubtless, was the secret of the witchcraft of old times. The magician did not perform a miracle, but he acted upon the imagination of his dupe till it seemed to the latter that a miracle had been performed. Some day, of course, there came along a hard-headed person, who could not be hypnotised, and therefore saw no signs or wonders; but women, who are the best subjects for hypnotism, are also the class who most like dabbling in the mysterious and unknown; and so the wizard would find no lack of clients. The most serious point in connection with this new discovery is its possible relation to crime. While in the hypnotic trance, a subject may do any evil suggested to him without intent or consciousness. Such crimes have before this been made the subject of romances, in which readers of gloomy imagination may revel at will; but if "the fairy tales of science" are to become the hard, realistic stories of the police-court, grave issues are at stake; and hypnotism, like lunacy, will be looked upon as a disease of which the law must take cognisance.

**A Salt Water Supply for Edinburgh.**

Edinburgh is taking a step in the right direction. A committee has been appointed to report on the practicableness and cost of having a salt water supply brought into the city. Edinburgh has the Firth of Forth practically at the door; its northern suburbs extend to the water's edge; and without excessive cost it could do as other towns, not more favourably situated, are doing, and provide every householder who desired it with a large and constant supply of salt water. The thing is done at North Shields, and North Shields is one of the healthiest towns in England. There the drains are abundantly flushed with salt water, which is also used for watering the streets. This gives the macadamised roads and causeways a bright, sparkling, frost-like appearance in summer, which is refreshing in itself. In the centre of the town are public baths abundantly supplied with salt water—an unspeakable boon to those of the inhabitants to whom a yearly visit to the seaside is an unattainable luxury. Householders can connect their houses with the main supply on payment of £1 a-year for baths, and 2s. 6d. for other sanitary purposes. And all this is maintained at a cost of £260 per annum. The initial expense is, of course, considerable, but North Shields has not found it unendurable, but well counterbalanced by the benefit of a supply of salt water. A pumping engine is placed in a building on the beach. This engine, which cost £500, is capable of raising 9,000 gallons per hour to a height of 150 feet, and sends the water inland to the reservoirs, a distance of nearly two miles. The reservoirs hold 250,000 gallons, and as the engine works fourteen hours a-day in summer, and little less in winter, they are kept always amply filled. The salt water supply was an experiment, but one which seems to have been amply justified by its success. This North Shields has done; this Edinburgh intends to do; and we hope that the committee now appointed will not let the grass grow beneath their feet, but will soon establish the necessary buildings and machinery. In a sea-girdled country like ours, with the energy and resource on which we pride ourselves, it should not be very difficult to provide all the towns that are situated at all near the coast with a sufficient supply of salt water, a thing which would add greatly to the health of their inhabitants and to their general amenity.

**The Phonograph.**

The phonograph adds a new terror to life. Mr. Edison threatens to make it so cheap that, as the advertisements say, "no household should be without it." What we shall all do when the dreadful things become articles of daily domestic use it is impossible to say. No husband will feel safe in scolding his wife any longer if the exuberances of his haste can be "turned on" against him in his calmer moments. Mrs. Caudle's pleasure in the delivery of her curtain lecture will be gone for ever when she knows that by a little management Mr. Caudle will be in a position to have it all repeated for the edification of the whole family at the breakfast table next morning. The new curate, with his high-pitched voice and his pretty little poetical vapidities, will be ready to rush down the pulpit stairs in mortal fright at the mere suspicion of a wicked schoolboy putting his hand anywhere near his waistcoat pocket. Dear Edwin will no longer venture to whisper his beautiful messages of love into the ears of his adored Angelina in the drawing-room, lest that dreadful imp, her brother, should have surreptitiously placed a couple of phonographs somewhere in the neighbourhood, and should bring out the conversation by instalments at any moment of the day. Nobody will feel in the least degree safe. Even a chairman of Quarter Sessions may sometimes forget that he is the representative of that science which is the perfection of reason, and give utterance to the "mere commonplaces" of social life like an ordinary man. What if the phonograph should catch up any of these, and at some moment when magisterial wisdom is at its solemnest should announce, in tones entirely bereft of magisterial pomp and dignity, that "the beefsteak is a little tough, Mrs. Trimley," or, "I think you had better get me a new woollen nightcap; the weather is getting cold"? Imagine the condition of the brother magistrates at such an occurrence, or the still more alarming state of the assembled bucolics, to whom the chairman is a far more majestic person than any queen or king could by any possibility be. There is only one further discovery wanted to make life absolutely not worth living. Let Mr. Edison invent a machine which will correctly read and reproduce our thoughts, and then we will all with one consent give it up in despair.

